# Z-Score Experience Replay in Off-Policy Deep Reinforcement Learning

**DOI:** 10.3390/s24237746

**Published:** 2024-12-04

**Authors:** Yana Yang, Meng Xi, Huiao Dai, Jiabao Wen, Jiachen Yang

**Affiliations:** The School of Electrical and Information Engineering, Tianjin University, Tianjin 300072, China; yangyana@tju.edu.cn (Y.Y.); daihuiao@tju.edu.cn (H.D.); wen_jiabao@tju.edu.cn (J.W.); yangjiachen@tju.edu.cn (J.Y.)

**Keywords:** deep reinforcement learning, off policy, priority experience replay, z-score

## Abstract

Reinforcement learning, as a machine learning method that does not require pre-training data, seeks the optimal policy through the continuous interaction between an agent and its environment. It is an important approach to solving sequential decision-making problems. By combining it with deep learning, deep reinforcement learning possesses powerful perception and decision-making capabilities and has been widely applied to various domains to tackle complex decision problems. Off-policy reinforcement learning separates exploration and exploitation by storing and replaying interaction experiences, making it easier to find global optimal solutions. Understanding how to utilize experiences is crucial for improving the efficiency of off-policy reinforcement learning algorithms. To address this problem, this paper proposes Z-Score Prioritized Experience Replay, which enhances the utilization of experiences and improves the performance and convergence speed of the algorithm. A series of ablation experiments demonstrate that the proposed method significantly improves the effectiveness of deep reinforcement learning algorithms.

## 1. Introduction

Reinforcement learning (RL) is commonly believed to have its roots in the behaviorist theories in psychology, where habitual behaviors that yield maximum benefits for an organism are gradually formed through a continuous process of being rewarded or punished by the environment. It was not until the late 20th century that RL began to receive attention from researchers and started to be rapidly developed, being considered as one of the core techniques for designing intelligent agents [1]. In the process of interacting with the environment, an agent selects actions based on the state in the current timestep, moves to the next state, and receives rewards. This process generates experiences, and the agent learns from these experiences to continuously improve its strategy and maximize cumulative rewards. Traditional reinforcement learning methods are suitable for discrete state spaces and cannot effectively handle problems in high-dimensional continuous state spaces. Linear value function approximation or nonlinear value function approximation can parameterize the relationship between states and value functions [2] to handle problems in high-dimensional state spaces. Linear value function approximation has good convergence guarantees, but it often requires manual feature extraction and has limited modeling capacity. Nonlinear value function approximation does not have strict convergence proofs but has a powerful modeling capacity. Tesauro [3] combined temporal difference (TD) learning with nonlinear value function approximation to propose the TD-Gammon algorithm. This algorithm uses a feedforward neural network to estimate the state value function and achieves top-level human performance in Backgammon.

RL interacts with the given environment via trial and error, adjusting the agent’s behavior strategy based on the received rewards [1]. This decision-making process, which resembles human experiential thinking and intuitive reasoning, has found widespread applications in the field of artificial intelligence [4,5]. However, as the complexity of application environments increases, the “curse of dimensionality” [6] limits the further development of RL. To better represent high-dimensional state spaces in complex task scenarios, DeepMind, the Google AI team, innovatively combined deep learning (DL) with RL, creating a new research focus called deep reinforcement learning (DRL) [7]. DRL combines the perceptual capability of DL with the decision-making ability of RL, enabling the learning of abstract representations from large-scale input data and using these representations to self-motivate and optimize problem-solving strategies [8]. Currently, DRL, with its end-to-end learning approach, has made significant progress in various domains such as game playing [9,10,11], robotics control [12,13,14], finance and trade [15,16,17], traffic control [18,19,20], and autonomous driving [21,22,23], where the performance of trained agents in these domains has reached or even surpassed human levels.

Unlike supervised learning and unsupervised learning, RL explores the environment through continuous interaction between an agent and its environment to acquire experience (samples). Based on this experience, the agent continuously updates its policy to find an optimal strategy that adapts to the environment. RL does not have a fixed dataset during the learning process; therefore, the agent needs to consume a significant amount of time and resources in order to obtain interactive experiences. In complex environments, particularly real-world environments such as autonomous driving, RL bears considerable risks and costs. Additionally, issues like wear and tear and response delays limit the amount of experience an agent can collect. How to effectively utilize limited experiences to train an intelligent agent with the best possible strategy has become a focal point for researchers both domestically and internationally. Storing and sampling experience samples is a key problem in off-policy deep reinforcement learning. During training, these methods use the error gradient backpropagation algorithm, which requires the training sample set to be independent and identically distributed (IID) or approximately IID to converge properly. However, samples exhibit strong temporal correlations. To eliminate sample correlation, scholars use an experience replay buffer to store and manage samples, and randomly sample from the buffer during training. They require a large-capacity experience replay buffer to store the samples. To populate the buffer, the agent needs to interact frequently with the environment. However, the cost of agent–environment interactions is expensive, not only in terms of time but also in terms of safety, controllability, and recoverability. Therefore, it is of great significance to study efficient sampling, reduce the number of agent–environment interactions, and improve the learning performance of the agent for deep reinforcement learning research. This emerging research area has quickly gained attention from a large number of researchers. So far, experience replay has become a prominent technique for improving the stability and convergence speed of off-policy deep reinforcement learning (DRL) algorithms.

The proposed method in this paper is adaptive, consistently adjusting to the changes in the deep network during training, resulting in a more reasonable estimation of sample priorities. Experimental results show that our approach enhances sample utilization, reduces interactions, and improves learning performance, ultimately leading to higher cumulative rewards.

## 2. Related Works

With the continuous development of deep reinforcement learning algorithms [24], many novel algorithms have been proposed by researchers worldwide to improve sample utilization efficiency [25]. The Dynamic Counterfactual Experience Replay (DCER) algorithm [26] addresses the issues of weak active exploration ability and suboptimal convergence in traditional deep reinforcement learning algorithms by creatively adding dynamic counterfactual experiences from both on-policy and off-policy contexts. This improves the efficiency of sample utilization by incorporating experiences that differ from the actual actions taken during sampling. The Quantum Experience Replay (QER) algorithm [27], inspired by quantum computing, adaptively selects training data from the experience replay buffer based on the complexity of each data sample and the number of replays, striking a balance between exploration and exploitation. The training data are represented in a quantum form and decayed over time, considering the relationship between temporal difference error and the importance of training data to ensure diverse selection of the training dataset. Kong et al. introduced a semi-normal sampling probability window in their work [28] to effectively balance the significance and novelty of sampled data. To expedite learning speed and improve performance while alleviating performance degradation during training, they prioritize more recent training data by assigning higher sampling probabilities to it. Ramicic et al. [29] utilize unsupervised learning techniques to categorize and sample training data into different context memory buffers, further reducing the correlation between training data samples.

With the continuous development of deep reinforcement learning algorithms, various mechanisms have emerged to guide the exploration of intelligent agents. Researchers worldwide have recognized the essence of exploration algorithms in deep reinforcement learning, which is to obtain as much information as possible while minimizing the search effort. Agrawal et al. [26] proposed a backward sampling algorithm combined with an optimistic optimization mechanism, providing a higher performance lower bound for solving optimal policies. The temporal difference ϵ-greedy algorithm [30] expands a single action into action sequences in the temporal dimension, replacing randomly selected actions in the ϵ-greedy algorithm. This algorithm improves the likelihood of convergence to global optima while retaining the simplicity of the ϵ-greedy algorithm. Amin et al. [31] combined the policy exploration process with the exploration trajectory data of the agent, generating trajectory data with local back-off properties in the state space, thus improving exploration efficiency and avoiding convergence to local optima. Jinnai et al. [32] used the deep coverage option method to autonomously discover task-agnostic options that encourage exploration, enhancing the exploration effectiveness of the intelligent agent and significantly reducing the time required for the agent to explore the entire state space. The active exploration method based on Bayesian models [33] utilizes the novelty of the probability transition distribution as a learning signal, applicable to both discrete and continuous environments. It calculates the Jensen–Shannon (JS) divergence and Jensen–Rényi (JR) divergence in prediction spaces of discrete and continuous environments, respectively. The agent only needs to maximize novelty measurements during the exploration process to make reasonable exploration behaviors.

The widely employed exploration strategy at present is the ϵ-greedy method. In this approach, during each decision-making process, there is a probability of 1-ϵ that the optimal action is selected based on the current policy, while there is a probability of ϵ that a non-optimal action is selected. This method addresses the trade-off between exploration and exploitation. Despite its simplicity, it is still widely used as an exploration strategy in some advanced reinforcement learning models. However, its performance is still subpar. The ϵ-greedy mechanism introduces some randomness in the action selection process, which can partially address the problem. However, it still fails to account for the uncertainty of the states. Traditional deep reinforcement learning algorithms commonly use uniform sampling from the experience replay pool to extract training data, sampling data in equal proportions. However, this method treats all data with equal importance, which is clearly different from the actual situation. Researchers have proposed mechanisms such as Emphasizing Recent Experience (ERE) [34] and Prioritized Experience Replay (PER) [35] to address this issue. However, these methods all have their respective limitations and may struggle to adapt to increasingly complex demands in the current scenario. This is because the probability of sampling a sample from the replay buffer is proportional to its storage priority, which is determined by its TD error, i.e., the difference between values when the sample was last involved in training. While the PER (Prioritized Experience Replay) algorithm improves the utilization efficiency of samples to some extent, in a replay buffer with a large number of samples, only a small fraction of samples can participate in the Q network update at each time step, while the remaining samples cannot be used for training. These samples have TD errors that do not change with the updates of the Q network, leading to the storage priority of samples not accurately reflecting the true distribution of TD errors in the replay buffer.

## 3. Method

### 3.1. Background

#### 3.1.1. Reinforcement Learning

**Figure 1:**
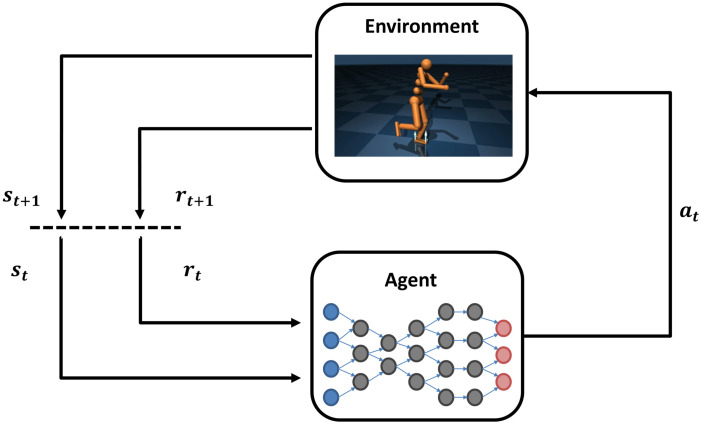
Relationships among the basic elements of reinforcement learning.

Reinforcement learning (RL) algorithms can be characterized as operating within the framework of Markov Decision Processes (MDPs). A Markov Decision Process (MDP) is defined as a tuple $\left(S,A,P,R,\gamma \right)$. Here, S represents the state space of the environment, A represents the set of possible actions in the environment, and P:S×A×S→[0,1] is the transition probability matrix. This matrix provides the probabilities of transitioning from one state $s_{t}$ to another state $s_{t+1}$ when taking action $a_{t}$. The function R:S×A→R defines the reward obtained from taking a certain action in a specific state. Lastly, the discount factor γ∈[0,1] determines the relative importance of future rewards compared to immediate rewards. In a given environment, the agent receives a state $s_{t}\in S$ at each time step *t*. The agent chooses an action $a_{t}$ from the set of possible actions A based on the policy π:S×A→[0,1]. After taking the chosen action, the given environment generates a new state $s_{t+1}\in S$ based on the transition function P. Then, the reward function R generates a reward $r_{t}$ and sends it to the agent. This process is illustrated in Figure 1. RL is a process of learning how to map environmental states to agent actions in order to maximize accumulated rewards [1]. The agent perceives the state of the environment, and then, using its own policy and the observed information, chooses and carries out an action. This action causes a transition in the environment’s state, and the environment provides rewards or penalties to the agent based on the action. The agent repeats this process until a predetermined terminal state is reached, concluding an iteration of the episode.

In RL, there are behavior policies and target policies. The behavior policy is the strategy the agent uses to select actions during the interaction with the environment, i.e., the policy used during the agent’s training process. The target policy refers to the action selection strategy adopted by the agent during the learning and optimization process using the experiences generated by the behavior policy. RL algorithms can be categorized into on-policy and off-policy algorithms based on whether the behavior policy and the target policy are the same.

#### 3.1.2. Priority Experience Replay

In order to eliminate the correlation between experiences, the experiences used for training in DQN satisfy the property of independent and identical distribution. DQN introduced the experience replay mechanism and designed an experience pool to store and manage experiences. During the training process, random sampling is used to select batches of experiences from the experience pool to renew parameters. This allows for the effective utilization of past experiences and further enhances the performance.

Prioritized Experience Replay is an effective method for estimating the priority of every sample. The samples are then selected and trained on the Q network based on their priority. The priority of a sample is controlled by its TD error. The larger the absolute value of the TD error of a sample, the larger the loss it causes in training the Q network, indicating that the sample provides more significant information.

Introducing randomness on the basis of priority during sampling is important. Sampling purely based on priority can decrease the diversity of the samples. Hence, in the PER algorithm, randomness is introduced to sample the experiences with probabilities proportional to their priorities, ensuring every sample has a chance to be selected. Additionally, the introduction of priorities changes the sample distribution compared to uniform sampling and introduces bias. The PER algorithm compensates for this bias by using importance sampling weights to adjust the gradient. During the calculation of the loss function gradient, the original gradient is multiplied by the importance sampling weights to update the parameters based on the compensated gradient. The PER algorithm offers great flexibility and can be widely combined with off-policy deep reinforcement learning methods.

#### 3.1.3. Z-Score

The z-score is a process of taking the difference between a number and the mean and then dividing it by the standard deviation. The z-score helps answer the question: “How many standard deviations is a given score away from the mean?” A score above the mean will have a positive z-score, and vice versa. The z-score is a technique used to determine the relative position of a particular score within a distribution.

By transforming the original scores of normally distributed data into z-scores, we can use a table of areas under the normal curve to determine the area between the mean and the z-score, indicating the percentile rank of the original score in the dataset. The sum of the squares of all z-scores in a sequence is equivalent to the total number of data points in that sequence. Additionally, the z-scores have a standard deviation and variance of 1, along with a mean of 0.

z-score indicates how far the score is from the mean and helps determine the position of the data point relative to the entire dataset. This process is called standardization. The formula for the transformation is as follows:

(1) $$z=\frac{X-\mu }{\sigma },$$

where *X* is the original data point to be standardized, μ is the mean value, and σ is the standard deviation value.

From the calculation of the z-score, it can be observed that the relationship between the original data and the mean is preserved, with the z-score of the mean equaling 0. Positive values indicate that the z-score is above the mean, while negative values indicate that it is below the mean. Furthermore, the magnitude of the difference between any original data point and the mean determines its position. Therefore, the z-score can represent both the relative size compared to other values and the position of a value. In statistics, the z-score is a critical indicator, and when the distribution of the original scores follows a normal distribution, converting all the original scores into z-scores results in a standard normal distribution.

### 3.2. Proposed Method

The problem with the PER algorithm is that the TD error values are not updated as promptly as the Q network; thus, it is unable to accurately reflect the priority of the samples. In each iteration, the Q network updates its own parameters, while the experience replay buffer only updates the TD error values of the batch samples involved in training, which account for only a portion of the experience replay buffer’s capacity.

This results in an inability of the TD error stored for the majority of the samples to accurately reflect the priority of the sample within the overall dataset. This further leads to a discrepancy between the storage priority and the actual distribution of the samples. This paper analyzes the impact of this deviation on learning and discusses how selecting samples based on the priority generated by the true or approximate TD error distribution can enable deep networks to converge to the optimal policy with higher efficiency.

**Figure 2:**
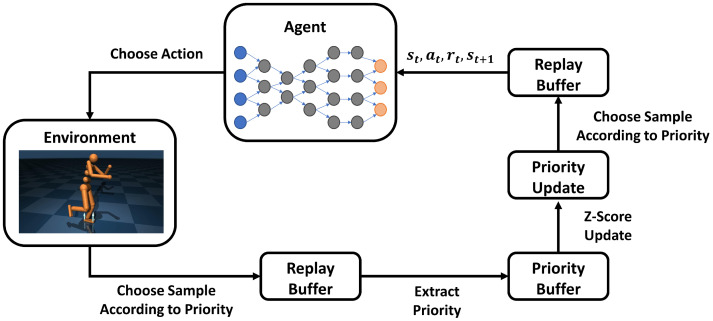
Overview.

This paper proposes a TD error-based z-score model to correct the storage priority of samples. By using the adjusted priority distribution for active sampling, the utilization efficiency can be improved. Figure 1 illustrates the overall structure of our approach, which mainly consists of the “bias model” and “priority correction” components. The priority correction needs to be performed at every time step, while the bias model is updated once every few time steps. The overview is shown in Figure 2.

Experience replay buffer is a memory system used in reinforcement learning to store and reuse past experiences. By sampling random past experiences during training, it helps break the correlation between consecutive samples, leading to more stable and efficient learning. In the PER algorithm, the experience replay buffer is represented as a collection of samples $E=e_{1},e_{2},\cdots ,e_{m}$, where each sample $e_{k}=\left(s_{k},a_{k},r_{k},s_{k+1}\right)$ is a tuple. The index of the sample $e_{k}$ in *E* is denoted as *k*, i.e., $e_{k}\in E$. In each iteration, the PER algorithm selects one batch sample from E for training. In this paper, the TD error value of a sample e from its last participation in training is referred to as the stored TD error and denoted as $\delta_{e}$. Let the current time step be *t*, and the time step of *e* last participation in training be $t_{e}$; then, $\delta_{e}$ is calculated using Equation (2).

(2) $$\begin{array}{c} \delta^{\left(i\right)}=r^{\left(i\right)}+\gamma Q\left(s^{\left(i\right)},\arg\;\max Q\left({s\prime }^{\left(i\right)},a;\theta_{t-\tau^{\left(i\right)}}\right);\right.\\ \left.\theta_{t-\tau^{\left(i\right)}}^{-}\right)-Q\left(s^{\left(i\right)},a^{\left(i\right)};\theta_{t-\tau^{\left(i\right)}}\right), \end{array}$$

where $\theta_{t-\tau^{\left(i\right)}}$ and $\theta_{t-\tau^{\left(i\right)}}^{-}$ are the parameters of the Q network and target network, respectively.

From the equation above, it can be observed that the parameter θ used in the calculation of the stored TD error is not a current parameter of the Q network but a parameter from a previous time step. Within z time steps, the Q network undergoes ρ iterations of parameter updates, while the stored TD error values remain unchanged. This results in a mismatch between the stored TD error of the samples and the current Q network, with a larger ρ leading to a higher degree of mismatch. When new samples are added to the experience replay buffer, for every time step participated in training, the priority of the corresponding sample is set to 1, and for samples that did not participate in training in that time step, the priority is incremented by 1. Since new samples overwrite old ones once the experience replay buffer is full, the values of all the samples range from 1 to the total number of samples in the buffer. Samples with higher values in the experience replay buffer have not been involved in training for a long time and their TD error values have not been updated periodically. Therefore, the stored priority is highly mismatched with the current Q network.

Introducing an ideal calculation method for the TD error, referred to as the “true” TD error, denoted as δ, requires calculating the distribution of δ by feeding stored samples into both the Q network and the target Q network. This involves updating the TD error values of these samples, including those that participated in the previous training session. In this calculation method, the TD error of any sample is computed using the most recent parameters of the Q network and the target Q network. This is represented by Equation (3).

(3) $$\begin{array}{c} \delta_{real}^{\left(i\right)}=r^{\left(i\right)}+\gamma Q\left(s^{\left(i\right)},\arg\;\max Q\left({s\prime }^{\left(i\right)},a;\theta_{t}\right);\right.\\ \left.\theta_{t}^{-}\right)-Q\left(s^{\left(i\right)},a^{\left(i\right)};\theta_{t}\right), \end{array}$$

where θ and $\theta^{-}$ are parameters of the Q network and the target Q network, respectively.

Let $p^{\left(i\right)}$ and $p_{real}^{\left(i\right)}$ represent the stored priority and true priority of a sample, respectively, corresponding to the stored TD error value δ and the true TD error value $\delta_{real}$. They are calculated using Equations (4) and (5).

(4) $$p^{\left(i\right)}=\left(\right|\delta^{\left(i\right)}{\left|+\epsilon \right)}^{\alpha },$$

(5) $$p_{real}^{\left(i\right)}=\left(\right|\delta_{real}^{\left(i\right)}{\left|+\epsilon \right)}^{\alpha },$$

where ϵ and α are both constant values.

The distribution of δ cannot accurately reflect the true state. When δ is large, the probability of δ sample being selected is high, and the value of *z* is small, meaning that the Q network does not change significantly during this period and the priority deviation is small. In such cases, the stored priority can be used as an approximate estimate of the true priority. However, when δ is small, the value of *z* is large, and the Q network undergoes significant parameter changes over *z* time steps, resulting in a deviation between the stored priority and the true priority distribution. In this situation, relying solely on the stored priority based on δ cannot accurately reflect the state of the experience replay buffer.

Although $p_{real}^{\left(i\right)}$ is a more ideal distribution of priorities, it can only be used in small-scale problems. In large-scale reinforcement learning, calculating the distribution of pa requires feeding all the samples into the Q network and the target Q network at each time step. Due to the large number of time steps and the large capacity of the experience replay buffer, this computation incurs significant time and storage costs. This paper proposes a TD error-adaptive correction method based on the z-score method, thereby obtaining an estimation of the true priority. The paper does not directly calculate the true priority $p^{n}$, but instead achieves the sampling priority that follows the updates of the Q network with minimal cost.

The samples extracted from the experience replay buffer are the inputs to the z-score model. The computed results from the z-score model are the outputs of the model. In general, the lower the stored priority *p* of a sample, the higher the priority deviation. To make the model more generalizable across different environments and training stages, the definition of the stored priority is modified based on Equation (4). The stored priority is normalized using the maximum value of the sample priorities and denoted as $p^{new}$. The calculation method of $p^{new}$ is as follows.

(6) $$p^{new\_(i)}=\frac{p^{\left(i\right)}-\mu \left(p^{\left(i\right)}\right)}{\sigma (p^{\left(i\right)})}.$$

The replay period of a sample is represented by the number of time steps since its last participation in training. The distribution of the replay period is inverted. As training progresses, samples are continuously added to the buffer. New samples and recently trained samples have a replay period of z=1. The values of all the samples range from 1 to the total number of samples in the buffer, since all samples fall within the range of the buffer’s capacity. Similarly, using the maximum value of the entire set of samples, the corresponding replay period is defined as τ and denoted as $\tau^{new}$.

(7) $$\tau^{new\_(i)}=\frac{\tau^{\left(i\right)}-\mu \left(\tau^{\left(i\right)}\right)}{\sigma (\tau^{\left(i\right)})}.$$

Assuming that the algorithm updates the old sample at each time step and the batch size is the same as the parameters of the original Q network update, this is equivalent to performing an additional forward inference of the Q network at each step. The calculation of z-score only involves the calculation of mean and variance, and the complexity of the calculation process is negligible compared with Q network reasoning.

Through complexity analysis, the proposed z-score PER algorithm in this paper can correct the stored priority in each iteration of the Q network with minimal time and space costs. The corrected priority is then used to select samples.

The pseudo-code corresponding to the algorithm is shown in Algorithm 1.
**Algorithm 1** z-score experience replay algorithm.**Require:** Minibatch *k*, step-size η, replay period *K* and size *N*, exponents α and β, and budget *T*.
  1:Initialize replay memory H=∅, $p_{1}=1$  2:Observe $S_{0}$ and choose $A_{0}∼\pi_{\theta }\left(S_{0}\right)$  3:**for** t=1 to *T* **do**  4:    Choose action $A_{t}∼\pi_{\theta }\left(S_{t}\right)$ and observe $S_{t},R_{t},\gamma_{t}$  5:    Store transition $\left(S_{t-1},A_{t-1},R_{t},\gamma_{t},S_{t}\right)$ in H with maximal priority $p_{t}=\max_{i<t}p_{i}$  6:    **if** t≡0modK **then**  7:        Compute TD error according to Equation (3) for new data and old data sampled from the replay period distribution $\tau^{new\_(i)}$  8:        Update priority according to Equations (6) and (7)  9:        Sample transition $j∼P\left(j\right)=p_{j}^{\alpha }/\sum_{i}p_{i}^{\alpha }$10:        Compute importance sampling weight $w_{j}={\left(N\cdot P\left(j\right)\right)}^{-\beta }/\max_{i}w_{i}$11:        Update transition priority $p_{j}\leftarrow \left|\delta_{j}\right|$12:        Update weights $\theta \leftarrow \theta +\eta \cdot w_{j}\cdot \delta_{j}\cdot \nabla_{\theta }Q\left(S_{j-1},A_{j-1}\right)$13:        Update the other SAC loss function14:    **end if**15:**end for**


## 4. Experiments

### 4.1. Experimental Environments and Settings

**Figure 3:**
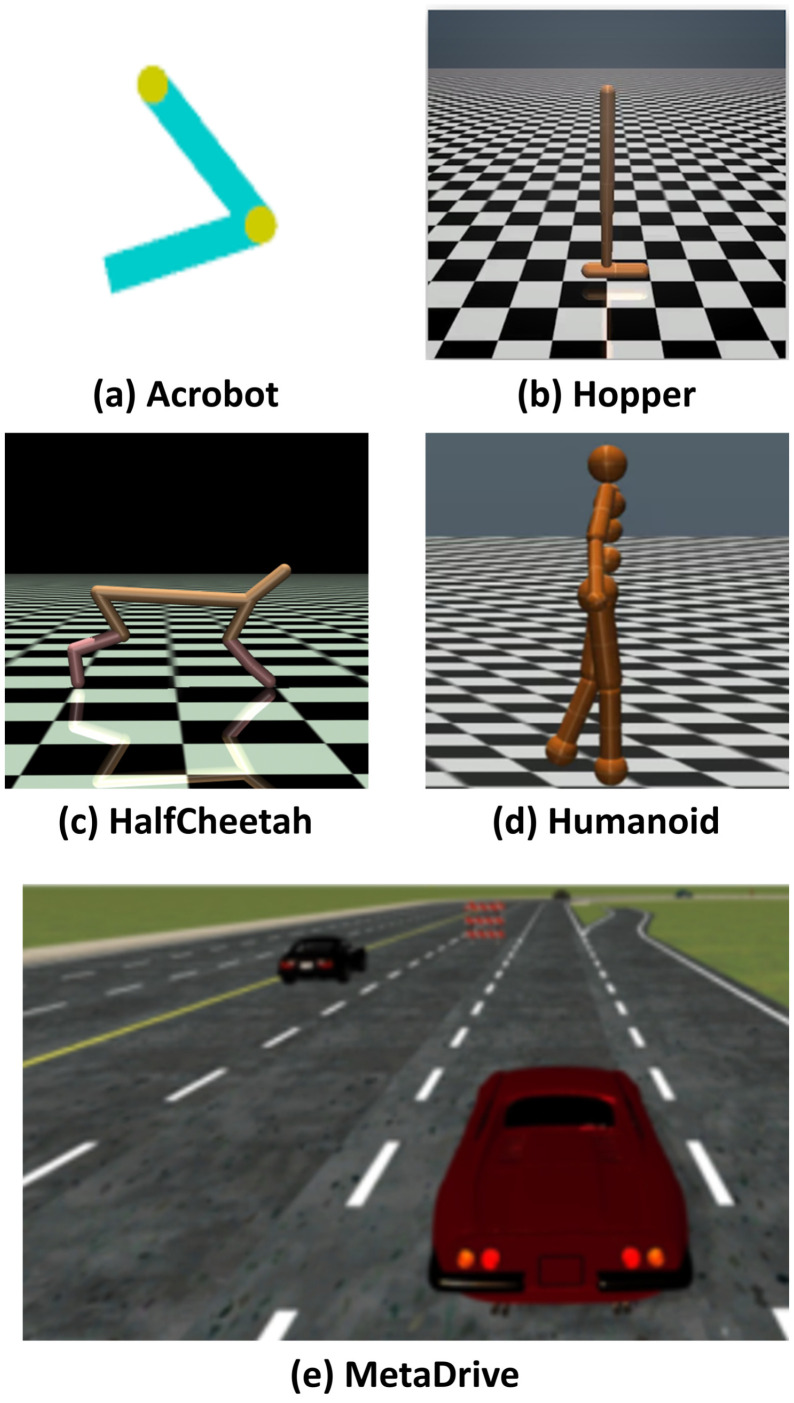
A schematic diagram of the experimental environments.

The experiments in this paper were conducted on a server equipped with i7-12700k CPUs (Intel, Santa Clara, CA, USA) and RTX 3080Ti GPUs (NVIDIA, Santa Clara, CA, USA). The chosen environments include one based on OpenAI Gym [36] called Acrobot, and three based on Mujoco [37] called Hopper, HalfCheetah, and Humanoid. The subsequent overview provides a brief description of these environments. Diagrams depicting these environments are shown in Figure 3.

**Acrobot**: The Acrobot environment offered by OpenAI Gym simulates a two-link pendulum system with a motor at the joint. The aim of this task is to swing the lower link of the pendulum above a predefined threshold by applying torque at the joint. The state of the Acrobot environment consists of the angular positions and velocities of the two links. The agent can choose from three discrete actions: applying a negative torque, applying a positive torque, or not applying any torque. The objective for the agent is to learn an optimal policy that efficiently applies torque to swing the lower link to the target height while minimizing energy consumption. In this environment, a reward of −1 is assigned for each time step until the task is successfully completed. Upon reaching the target height, a reward of 0 is given. The episode terminates if the pendulum drifts to a position where it cannot swing up anymore. The dynamics of the Acrobot environment adhere to the laws of physics, including gravity, inertia, and the applied torque. The agent needs to understand these factors and their interactions to effectively manipulate the pendulum and achieve the desired goal. By employing reinforcement learning algorithms, agents can be trained to learn policies that balance actions and explore different strategies to successfully complete the task. The Acrobot environment presents a challenging and engaging scenario for reinforcement learning agents to master the control of a pendulum-like system and accomplish a specific objective.

**Hopper**: The Hopper environment within the MuJoCo simulator is a realistic and dynamic simulation of a two-legged robot known as a “hopper”. The primary objective of the Hopper task is to teach the robot how to hop and maintain balance while advancing forward. The state of the Hopper environment encompasses various attributes such as the hopper’s body and joint position, velocity, and angular position. The action space consists of continuous actions that control the amount of torque applied to the robot’s joints. Torque here includes Torque applied on the thigh rotor, Torque applied on the leg rotor, and Torque applied on the foot rotor. By applying the appropriate torques, the agent aims to learn a policy that enables the hopper to perform hopping motions and propel itself forward. To incentivize the agent to achieve its goal, the Hopper environment incorporates a reward signal, which is determined based on the hopper’s forward velocity. Increasing velocity incurs a positive reward, while falling or deviating from an upright position results in a negative reward. The episode concludes if the hopper falls or moves too slowly. The dynamics of the Hopper environment are simulated using a physics engine that considers factors like gravity, joint constraints, and body dynamics. To master the hopping behavior, the agent needs to employ techniques like balance control, energy management (by setting action penalties), and leg movement coordination. Through reinforcement learning algorithms, the agent can be trained to explore and discover effective policies within the Hopper environment. The ultimate objective is to find a policy that achieves high forward velocity while sustaining balance throughout the hopping motion. Overall, the Hopper environment presents a challenging and dynamic training ground for reinforcement learning agents to acquire the necessary locomotion skills to control a two-legged robot in tasks involving hopping and maintaining balance while moving forward.

**HalfCheetah**: The MuJoCo simulator features the HalfCheetah environment, which is a dynamic simulation of a robot resembling a half-actuated cheetah. The primary objective of the HalfCheetah task is for the robot to acquire locomotion skills and optimize its forward velocity while also maintaining balance. The state of the HalfCheetah environment encompasses details about the positions, velocities, and angles of different body parts of the robot. The action space comprises continuous actions that control the torques applied to the robot’s joints. By skillfully applying torques, the agent aims to learn a policy that enables the HalfCheetah to swiftly move forward. In the HalfCheetah environment, the agent receives a reward signal based on its forward speed. It is rewarded positively for increasing velocity and penalized for instability or deviations from an upright position. Episodes conclude if the robot falls or moves too slowly. The dynamics of the HalfCheetah environment are simulated using a physics engine that accounts for factors like gravity, inertia, and friction. The agent needs to employ techniques such as balance control, leg movement coordination, and energy management to excel in the task. Reinforcement learning algorithms can be employed to train the agent to explore and discover effective policies within the HalfCheetah environment. The goal is to find a policy that achieves high forward velocity while maintaining balance. In conclusion, the HalfCheetah environment provides a challenging setting for training reinforcement learning agents to acquire locomotion skills. By navigating the balance between speed and stability, the agent seeks to control the half-actuated cheetah-like robot and optimize its forward velocity.

**Humanoid**: The MuJoCo simulator offers the Humanoid environment, a sophisticated and lifelike simulation of a humanoid robot. The Humanoid task involves training the robot to acquire diverse locomotion skills, such as walking, running, and executing dynamic movements. The state of the Humanoid environment comprises information on the positions, velocities, and orientations of the robot’s various body parts. The action space consists of continuous actions controlling the torques applied to the robot’s joints. The agent’s objective is to learn policies that generate coordinated joint movements to achieve efficient and stable locomotion for the humanoid. In the Humanoid environment, rewards are determined based on the robot’s behavior and progress towards the desired task. The specific reward design depends on the particular task, but it typically includes factors like forward velocity, energy efficiency, or task-specific objectives like reaching a target or following a trajectory. The dynamics of the Humanoid environment are simulated using a physics engine that considers elements such as gravity, inertia, friction, and contact forces. The agent must master techniques like balance control, gait generation, and energy optimization to execute skilled locomotion. Reinforcement learning algorithms can be employed to train an agent to explore and discover effective policies within the Humanoid environment. The ultimate goal is to find a policy that enables the humanoid robot to perform various locomotion tasks with stability, efficiency, and adaptability. In conclusion, the Humanoid environment offers a challenging and realistic platform for training reinforcement learning agents in acquiring intricate locomotion skills.

**MetaDrive**: MetaDrive is a comprehensive simulation environment specially developed for the purposes of autonomous driving research and development. It provides a realistic and dynamic urban environment equipped with advanced control options, making it highly suitable for testing a wide range of autonomous driving algorithms. In the MetaDrive environment, the action space is defined by a set of continuous control commands that the autonomous vehicle can execute. These commands allow for precise control over the vehicle’s movements, including steering angle, acceleration, and brake intensities. This continuous action space enables the development and evaluation of sophisticated autonomous driving policies. The state space in MetaDrive comprises a diverse set of observations that capture crucial information for autonomous driving. This includes data on vehicle positions, velocities, orientations, as well as details about the surrounding environment, such as traffic signals, road boundaries, and dynamic objects. The state space provides the necessary information for perception and decision-making processes within autonomous driving algorithms. In terms of the reward function, MetaDrive offers great flexibility, allowing users to define customized reward functions based on their specific research objectives. Typical reward functions may include considerations such as reaching a target destination efficiently, adhering to traffic regulations, maintaining safe distances from other vehicles, and minimizing fuel consumption. Researchers have the ability to design reward functions to align with their particular objectives and utilize them to guide the learning and optimization processes of their autonomous driving algorithms in the MetaDrive environment.

We use SAC, an off-policy deep reinforcement learning algorithm, as the baseline algorithm to validate the performance of our method in this study. The hyperparameters for SAC can be seen in Table 1 for experimental environments.

### 4.2. Results and Discussions

This section compares the performance and convergence speed differences among the following three algorithms. All experiments were conducted using five sets of random numbers randomly selected, and the final experimental results were averaged.

- SAC: The original SAC algorithm, without any Prioritized Experience Replay mechanism.
- SAC-PER: An SAC algorithm with the Prioritized Experience Replay mechanism.
- SAC-ZS-PER: An SAC algorithm with the proposed Z-Score Prioritized Experience Replay mechanism.

**Figure 4:**
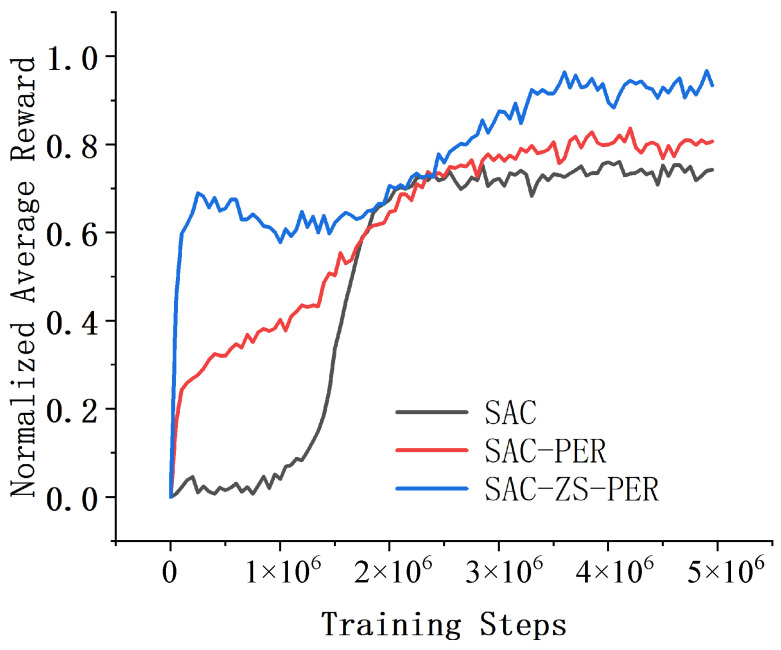
Comparison of results in the Acrobot environment.

In the Acrobot environment, we conducted a comparative analysis of three deep reinforcement learning algorithms, namely SAC, SAC-PER, and SAC-ZS-PER, in terms of their performance and convergence speed. The results, shown in Figure 4, revealed that SAC-ZS-PER demonstrated the highest performance and the fastest convergence speed, while SAC exhibited the lowest performance and the slowest convergence speed, with SAC-PER occupying an intermediate position. Upon comparing the performance and convergence speed of algorithms SAC, SAC-PER, and SAC-ZS-PER, it was observed that SAC-ZS-PER outperformed the others. It obtained the highest average cumulative reward and achieved the task goal more consistently compared to SAC and SAC-PER. This suggests that SAC-ZS-PER is more effective in learning the optimal policy for the Acrobot task, leading to better performance overall. In terms of convergence speed, SAC-ZS-PER also stood out by requiring the fewest episodes to reach the performance improvement threshold. This indicates that SAC-ZS-PER learns more efficiently and converges to an optimal policy faster than SAC and SAC-PER. The superior convergence speed of SAC-ZS-PER can be attributed to its architecture or the specific learning techniques employed, which facilitate quicker exploration and exploitation of the environment. SAC, on the other hand, showed the lowest performance and the slowest convergence speed among the three. This indicates that the original version of SAC might be less suitable or less effective for solving the Acrobot task. As a result, it struggles to achieve satisfactory performance and requires more episodes to learn and improve. SAC-PER, although not as proficient as SAC-ZS-PER, demonstrated a moderate level of performance and convergence speed. It falls between SAC and SAC-ZS-PER in terms of both metrics, suggesting a relatively balanced trade-off between the two.

**Figure 5:**
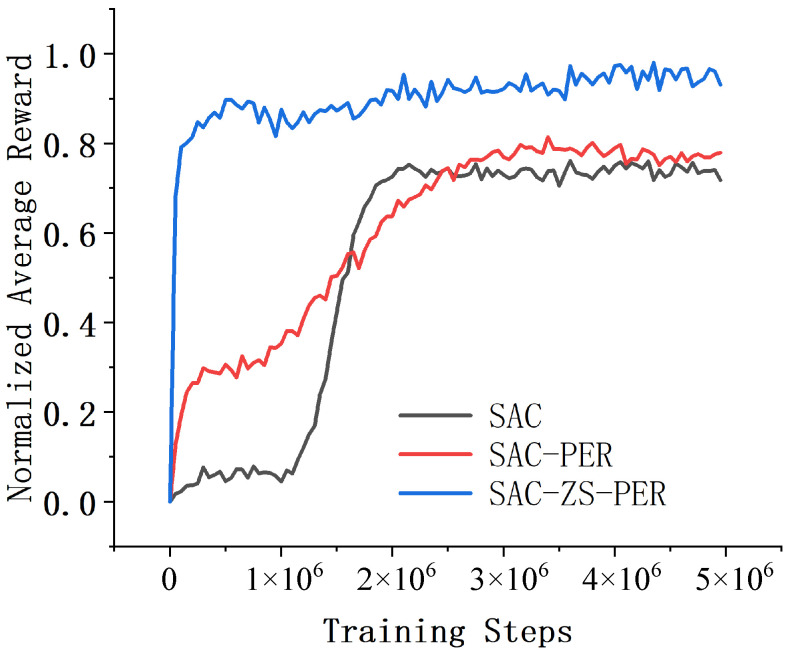
Comparison of results in the Hopper environment.

In the Hopper environment, we compared the performance and convergence speed of SAC, SAC-PER, and SAC-ZS-PER. The results are shown in Figure 5. These results revealed that SAC-ZS-PER exhibited the highest performance and the fastest convergence speed, while SAC demonstrated the lowest performance and the slowest convergence speed, with SAC-PER falling in between. Upon comparing the performance and convergence speed of algorithms SAC, SAC-PER, and SAC-ZS-PER, it was evident that SAC-ZS-PER outperformed the others. It achieved the highest average reward, signifying that it was the most successful in finding appropriate control policies to enable effective hopping motions. This suggests that SAC-ZS-PER exhibits strong capabilities in exploring and learning the complex dynamics of the Hopper environment, resulting in superior performance. Furthermore, SAC-ZS-PER demonstrated the fastest convergence speed among the three. SAC-ZS-PER required the fewest episodes to reach a satisfactory level of hopping performance. This highlights its ability to efficiently learn and adapt, enabling it to reach optimal policies at a faster pace. On the other hand, SAC exhibited the lowest performance and the slowest convergence speed. SAC-PER is positioned between SAC and SAC-ZS-PER in terms of both performance and convergence speed. Although not as proficient as SAC-ZS-PER, it demonstrates a moderate level of performance and convergence speed. This suggests that the selected architecture or learning techniques in SAC-PER achieve a reasonable balance between performance and convergence speed. It achieves satisfactory hopping motions but may require a longer training period compared to SAC-ZS-PER.

**Figure 6:**
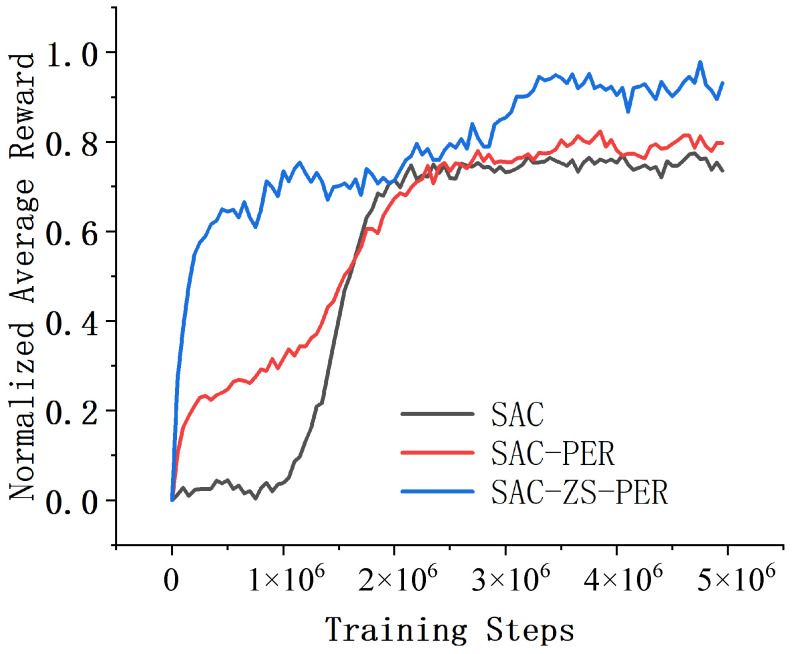
Comparison of results in the HalfCheetah environment.

In the HalfCheetah environment, a series of experiments were conducted to evaluate the performance and convergence speed of SAC, SAC-PER, and SAC-ZS-PER. The results, displayed in Figure 6, demonstrate that SAC-ZS-PER exhibited the highest performance and the fastest convergence speed, while SAC showcased the lowest performance and the slowest convergence speed, with SAC-PER falling in between. The analysis of the algorithms’ performance and convergence speed revealed that SAC-ZS-PER outperformed the others. It achieved the highest average reward, showcasing its superior ability to learn optimal policies and achieve rapid forward motion in the HalfCheetah environment. SAC-ZS-PER demonstrated a robust understanding of the environment’s dynamics, resulting in highly efficient locomotion patterns. Moreover, SAC-ZS-PER displayed the fastest convergence speed among the three algorithms. In this context, it measures the number of episodes required for the algorithm to attain a desirable level of forward motion. SAC-ZS-PER required the fewest episodes to achieve satisfactory locomotion performance, highlighting its ability to swiftly adapt and learn effective policies. On the contrary, SAC exhibited the lowest performance and the slowest convergence speed in the HalfCheetah environment. SAC-PER, positioned between SAC and SAC-ZS-PER, demonstrated moderate performance and convergence speed. Although not as impressive as SAC-ZS-PER, its performance was reasonable, and it achieved a satisfactory level of forward motion.

**Figure 7:**
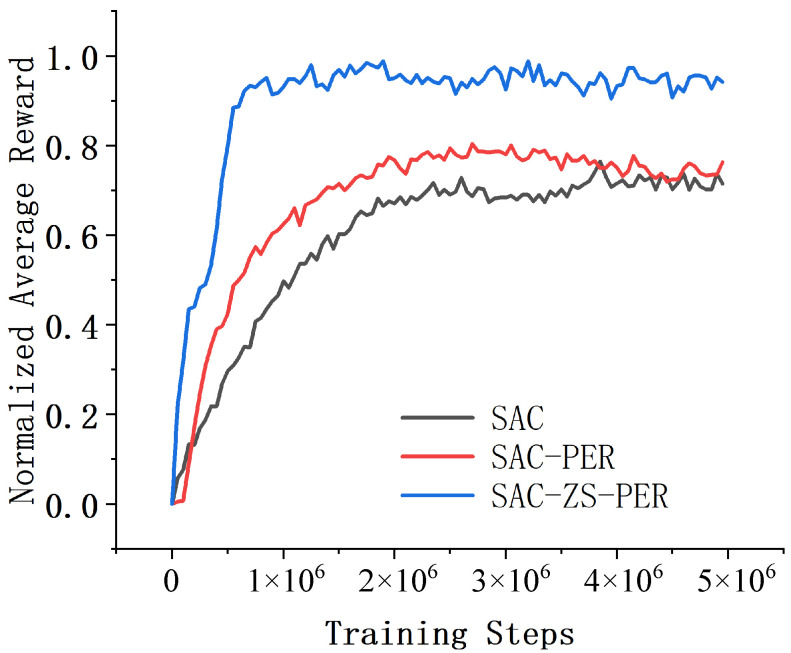
Comparison of results in the Humanoid environment.

In the Humanoid environment, SAC, SAC-PER, and SAC-ZS-PER were assessed in terms of their performance and convergence speed. The results revealed that SAC-ZS-PER exhibited the highest performance and the fastest convergence speed, while SAC demonstrated the lowest performance and the slowest convergence speed, with SAC-PER falling in between. Upon the comparison of the algorithms’ performance and convergence speed, SAC-ZS-PER stood out as the top performer. These results are depicted in Figure 7. It achieved the highest average reward, indicating its proficiency in learning complex walking patterns and maintaining balance in the Humanoid environment. SAC-ZS-PER displayed a robust understanding of the intricate dynamics involved and consistently achieved superior walking performance. Furthermore, SAC-ZS-PER demonstrated the fastest convergence speed among the three algorithms. SAC-ZS-PER required the fewest episodes to reach this level, highlighting its ability to efficiently learn and adapt to the demanding Humanoid environment. In contrast, SAC exhibited the lowest performance and the slowest convergence speed in the Humanoid environment. SAC-PER, positioned between SAC and SAC-ZS-PER, demonstrated moderate performance and convergence speed. Although not as exceptional as SAC-ZS-PER, it achieved a reasonable level of walking performance and balance control.

**Figure 8:**
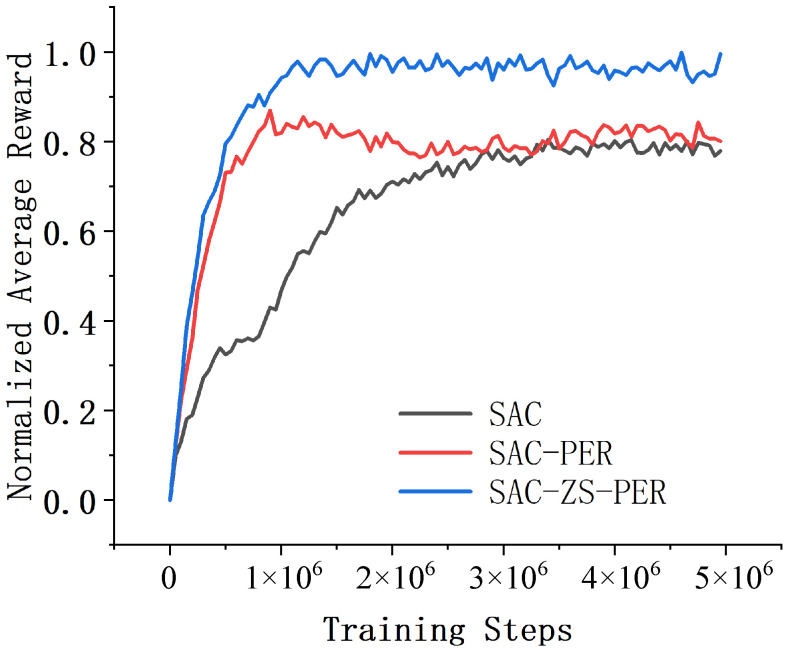
Comparison of results in the MetaDrive environment.

In the MetaDrive environment, we conducted a comparative analysis of three deep reinforcement learning algorithms: SAC, SAC-PER, and SAC-ZS-PER, to assess their performance (measured as the return value) and convergence speed. The results revealed a clear distinction in the capabilities of these algorithms. They are demonstrated in Figure 8. SAC-ZS-PER emerged as the top performer, showcasing the highest performance and the fastest convergence speed. SAC, on the other hand, demonstrated the lowest performance and the slowest convergence speed, while SAC-PER occupied a moderate position. SAC-ZS-PER showcased exceptional performance in the MetaDrive environment, achieving the highest average completion time and a remarkable success rate in reaching destinations. This indicates that it successfully learned to navigate the road scenarios efficiently, reaching destinations with minimal delays and without violating traffic rules. SAC-ZS-PER demonstrated strong decision-making capabilities and effective control strategies, resulting in superior performance. Additionally, SAC-ZS-PER displayed the fastest convergence speed among the three algorithms. SAC-ZS-PER required the fewest episodes or iterations to converge to a satisfactory level of performance in MetaDrive. Its ability to rapidly extract meaningful information from the environment and optimize its decision-making process allowed for fast convergence and exceptional performance. In contrast, SAC exhibited the lowest performance and the slowest convergence speed in the MetaDrive environment. SAC struggled to achieve satisfactory completion times and success rates, requiring more iterations to improve its performance. SAC-PER showcased moderate performance and convergence speed in MetaDrive. It achieved completion times and success rates that were intermediate between SAC and SAC-ZS-PER.

## 5. Conclusions

In off-policy reinforcement learning algorithms, methods based on Prioritized Experience Replay do not accurately store the priority of samples during training. The stored priority does not accurately reflect the true distribution of TD errors in the experience replay buffer, thereby reducing the efficiency of utilizing a large number of samples. By using the true priority of z-score-processed samples to sample from the experience replay buffer, the utilization efficiency of the samples and the effectiveness of learning can be significantly improved.

Our proposed active sampling method based on z-score TD error-adaptive correction allows for the correction of priority biases in the storage of samples in off-policy reinforcement learning algorithms. By utilizing the z-score of the samples and the state of the Q network, we can estimate the true priority of the samples, approximating the true distribution with minimal cost. The bias model is updated in segments during the training process and is insensitive to the update frequency. Our proposed method provides an effective estimation of priority biases and significantly reduces the computational complexity. This method can be combined with the common off-policy reinforcement learning algorithm and replace the original preferential experience replay algorithm. It has a wide application prospect and is a plug-and-play algorithm module. In future research, calculating more efficient and accurate time difference error is an important means to further improve the effectiveness of priority experience playback algorithms.

## Figures and Tables

**Table 1:** Hyperparameters of SAC.

Hyperparameter	Value
Discount	0.99
Actor Learning Rate	$5\times 10^{-4}$
Critic Learning Rate	$5\times 10^{-4}$
Batch Size	128
Number of Hidden Layers	2
Hidden Layer Size	128
Training Timestep Number	$5\times 10^{6}$
α in PER	0.7
β in PER	0.3

## Data Availability

The data are available from the corresponding author on reasonable request.

## References

[1] Sutton R.S., Barto A.G. (2018). Reinforcement Learning: An Introduction.

[2] Bertsekas D.P., Tsitsiklis J.N. Neuro-dynamic programming: An overview. Proceedings of the 1995 34th IEEE Conference on Decision and Control.

[3] Tesauro G. (1994). TD-Gammon, a self-teaching backgammon program, achieves master-level play. Neural Comput..

[4] Li C., Cao L., Zhang Y., Chen X., Zhou Y., Duan L. (2017). Knowledge-based deep reinforcement learning: A review. Syst. Eng. Electron..

[5] Xi M., Dai H., He J., Li W., Wen J., Xiao S., Yang J. (2024). A lightweight reinforcement learning-based real-time path planning method for unmanned aerial vehicles. IEEE Internet Things J..

[6] Bellman R. (2010). Dynamic Programming.

[7] Wang Y., Tang C., Wang S., Cheng L., Wang R., Tan M., Hou Z. (2021). Target tracking control of a biomimetic underwater vehicle through deep reinforcement learning. IEEE Trans. Neural Netw. Learn. Syst..

[8] Yang J., Zhang Z., Xiao S., Ma S., Li Y., Lu W., Gao X. (2023). Efficient data-driven behavior identification based on vision transformers for human activity understanding. Neurocomputing.

[9] Silver D., Hubert T., Schrittwieser J., Antonoglou I., Lai M., Guez A., Lanctot M., Sifre L., Kumaran D., Graepel T. (2018). A general reinforcement learning algorithm that masters chess, shogi, and Go through self-play. Science.

[10] Heess N., Wayne G., Silver D., Lillicrap T., Erez T., Tassa Y. (2015). Learning continuous control policies by stochastic value gradients. Adv. Neural Inf. Process. Syst..

[11] Mnih V., Kavukcuoglu K., Silver D., Graves A., Antonoglou I., Wierstra D., Riedmiller M. (2013). Playing atari with deep reinforcement learning. arXiv.

[12] Zhang T., Tian R., Yang H., Wang C., Sun J., Zhang S., Xie G. (2022). From Simulation to Reality: A Learning Framework for Fish-Like Robots to Perform Control Tasks. IEEE Trans. Robot..

[13] Arnekvist I., Kragic D., Stork J.A. Vpe: Variational policy embedding for transfer reinforcement learning. Proceedings of the 2019 International Conference on Robotics and Automation (ICRA).

[14] Mees O., Merklinger M., Kalweit G., Burgard W. Adversarial skill networks: Unsupervised robot skill learning from video. Proceedings of the 2020 IEEE International Conference on Robotics and Automation (ICRA).

[15] Tsantekidis A., Passalis N., Tefas A. (2021). Diversity-driven knowledge distillation for financial trading using Deep Reinforcement Learning. Neural Netw..

[16] Taghian M., Asadi A., Safabakhsh R. (2022). Learning financial asset-specific trading rules via deep reinforcement learning. Expert Syst. Appl..

[17] Park H., Sim M.K., Choi D.G. (2020). An intelligent financial portfolio trading strategy using deep Q-learning. Expert Syst. Appl..

[18] Bouktif S., Cheniki A., Ouni A. (2021). Traffic signal control using hybrid action space deep reinforcement learning. Sensors.

[19] Du X., Wang J., Chen S., Liu Z. (2021). Multi-agent deep reinforcement learning with spatio-temporal feature fusion for traffic signal control. Proceedings of the Machine Learning and Knowledge Discovery in Databases. Applied Data Science Track: European Conference, ECML PKDD 2021.

[20] Gao R., Liu Z., Li J., Yuan Q. (2020). Cooperative traffic signal control based on multi-agent reinforcement learning. Proceedings of the Blockchain and Trustworthy Systems: First International Conference (BlockSys 2019).

[21] Zhou M., Yu Y., Qu X. (2019). Development of an efficient driving strategy for connected and automated vehicles at signalized intersections: A reinforcement learning approach. IEEE Trans. Intell. Transp. Syst..

[22] Hu J., Niu H., Carrasco J., Lennox B., Arvin F. (2020). Voronoi-based multi-robot autonomous exploration in unknown environments via deep reinforcement learning. IEEE Trans. Veh. Technol..

[23] Kendall A., Hawke J., Janz D., Mazur P., Reda D., Allen J.M., Lam V.D., Bewley A., Shah A. Learning to drive in a day. Proceedings of the 2019 International Conference on Robotics and Automation (ICRA).

[24] Yang J., Cheng C., Xiao S., Lan G., Wen J. (2023). High fidelity face-swapping with style convtransformer and latent space selection. IEEE Trans. Multimed..

[25] Yang J., Ma S., Zhang Z., Li Y., Xiao S., Wen J., Lu W., Gao X. (2024). Say No to Redundant Information: Unsupervised Redundant Feature Elimination for Active Learning. IEEE Trans. Multimed..

[26] Menglin L., Jing C., Shaofei C., Wei G. A new reinforcement learning algorithm based on counterfactual experience replay. Proceedings of the 2020 39th Chinese Control Conference (CCC).

[27] Wei Q., Ma H., Chen C., Dong D. (2021). Deep reinforcement learning with quantum-inspired experience replay. IEEE Trans. Cybern..

[28] Kong S.H., Nahrendra I.M.A., Paek D.H. (2021). Enhanced off-policy reinforcement learning with focused experience replay. IEEE Access.

[29] Ramicic M., Bonarini A. (2020). Correlation minimizing replay memory in temporal-difference reinforcement learning. Neurocomputing.

[30] Dabney W., Ostrovski G., Barreto A. (2020). Temporally-Extended *ϵ*-Greedy Exploration. arXiv.

[31] Amin S., Gomrokchi M., Aboutalebi H., Satija H., Precup D. (2020). Locally persistent exploration in continuous control tasks with sparse rewards. arXiv.

[32] Jinnai Y., Park J.W., Machado M.C., Konidaris G. Exploration in reinforcement learning with deep covering options. Proceedings of the International Conference on Learning Representations.

[33] Shyam P., Jaśkowski W., Gomez F. Model-based active exploration. Proceedings of the International Conference on Machine Learning, PMLR.

[34] Wang C., Ross K. (2019). Boosting soft actor-critic: Emphasizing recent experience without forgetting the past. arXiv.

[35] Schaul T., Quan J., Antonoglou I., Silver D. (2015). Prioritized experience replay. arXiv.

[36] Brockman G., Cheung V., Pettersson L., Schneider J., Schulman J., Tang J., Zaremba W. (2016). Openai gym. arXiv.

[37] Todorov E., Erez T., Tassa Y. Mujoco: A physics engine for model-based control. Proceedings of the 2012 IEEE/RSJ International Conference on Intelligent Robots and Systems.

